# Supplementary figures and images for: Testing of the therapeutic efficacy and safety of AMPA receptor RNA aptamers in an ALS mouse model
Source: Life Sci Alliance. 2022 Jan 12;5(4):e202101193. doi: 10.26508/lsa.202101193 (PMC8761490; doi:10.26508/lsa.202101193)

Fig 2B original autoradiographic data by BSA-2500

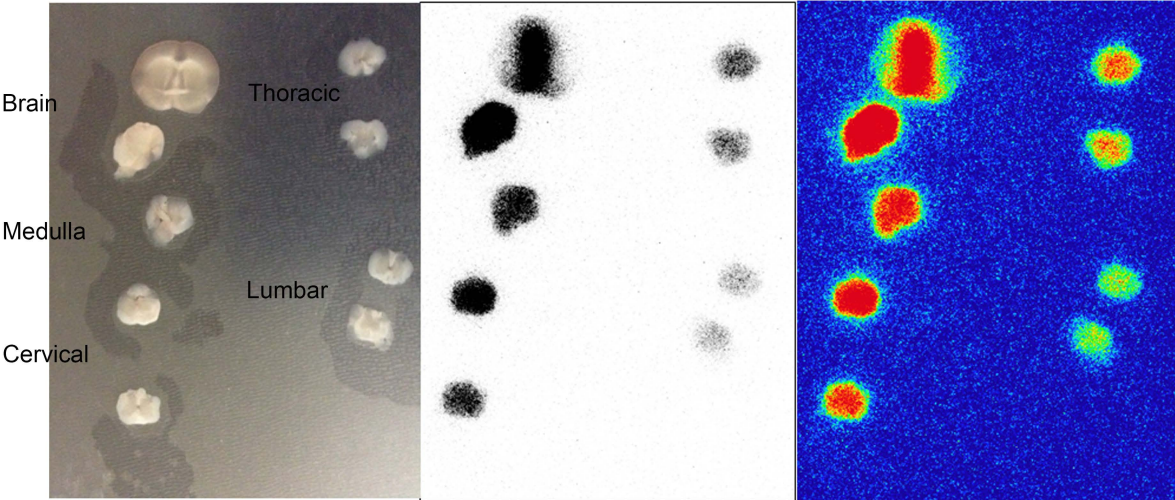

Fig 2D original electrophoretic blot

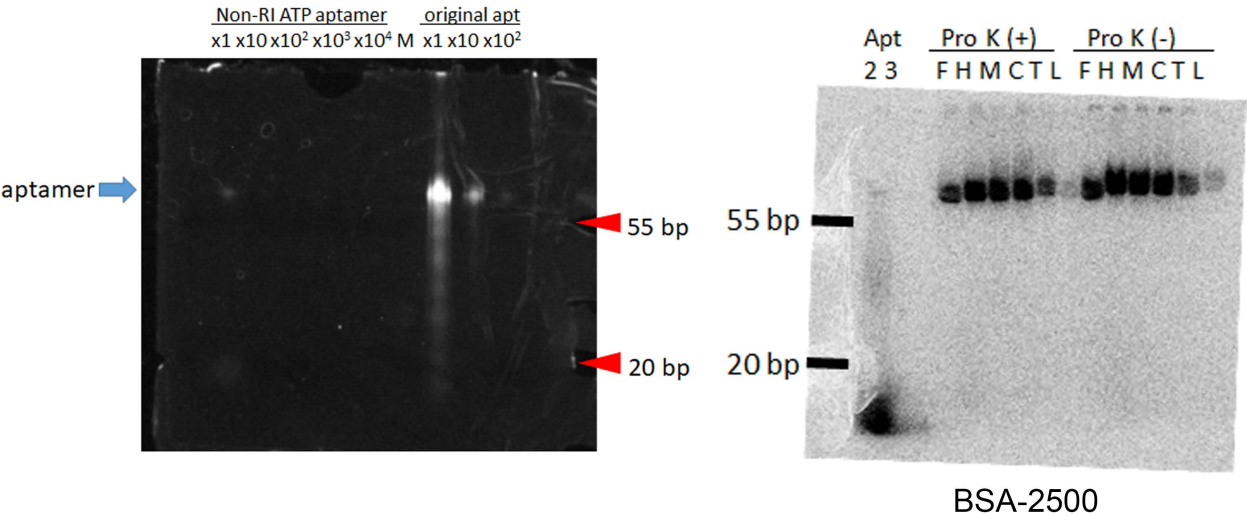

Supplement: Supplementary file 2 [file LSA-2021-01193_SdataF2.1.pdf]

Fig 3B original microscopic picture (scale bar = 20  $\mu\text{m}$ )

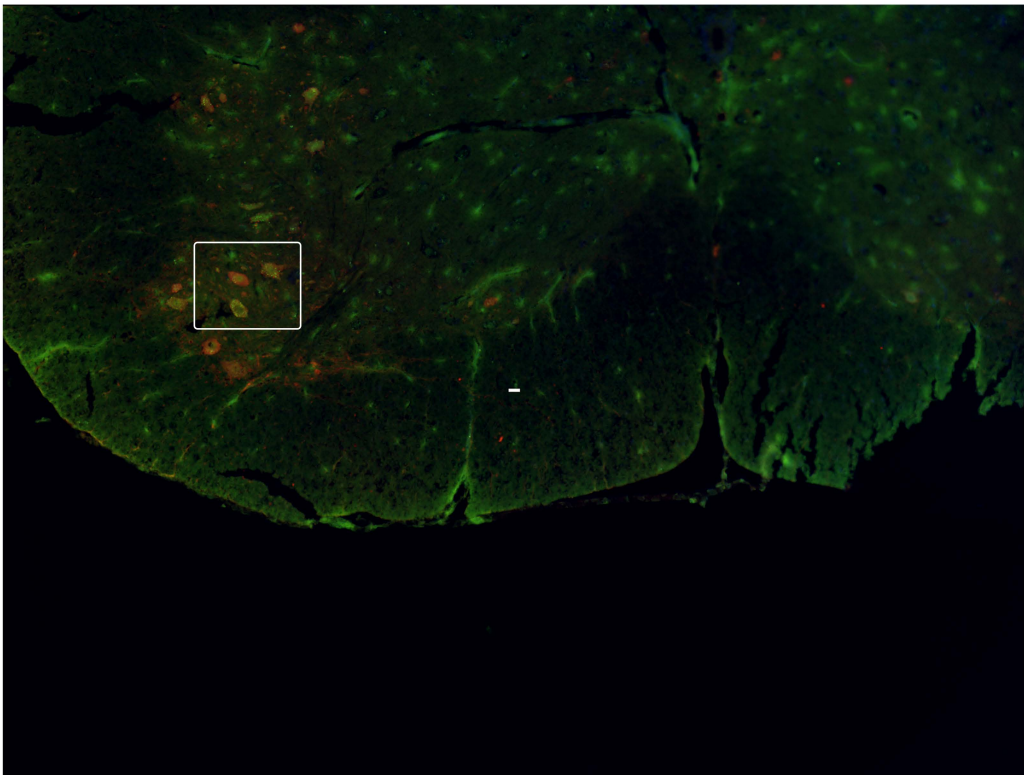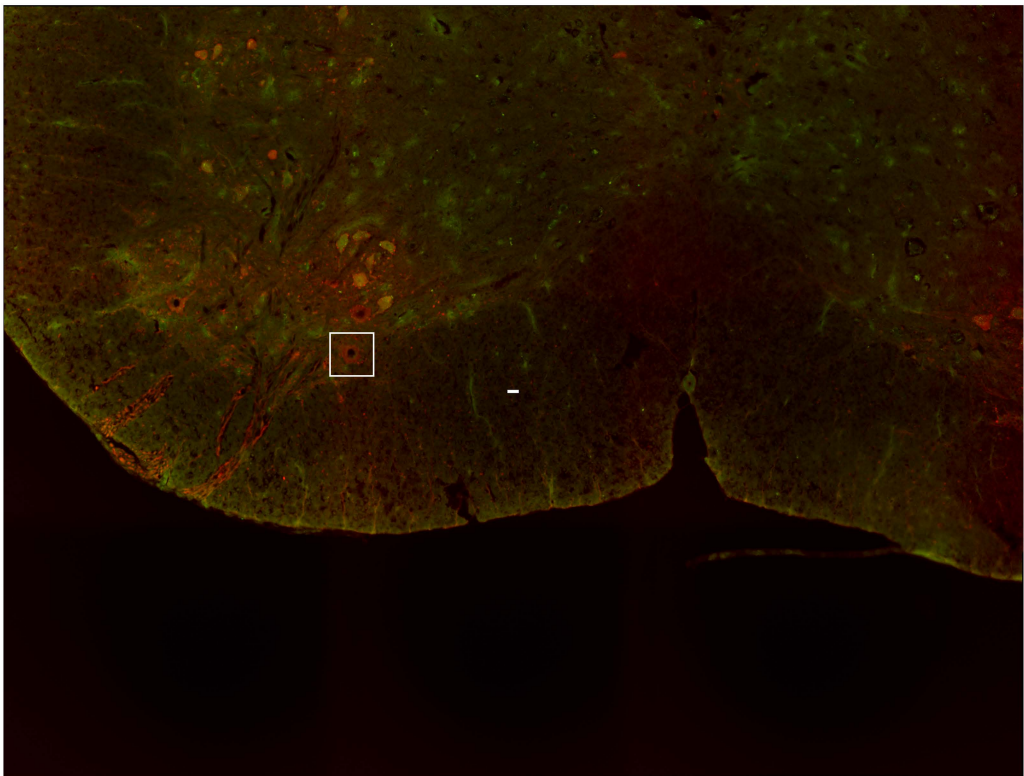

Supplement: Supplementary file 5 [file LSA-2021-01193_SdataF3.2.pdf]

Fig 4D original microscopic picture (scale bar = 20  $\mu$ m)

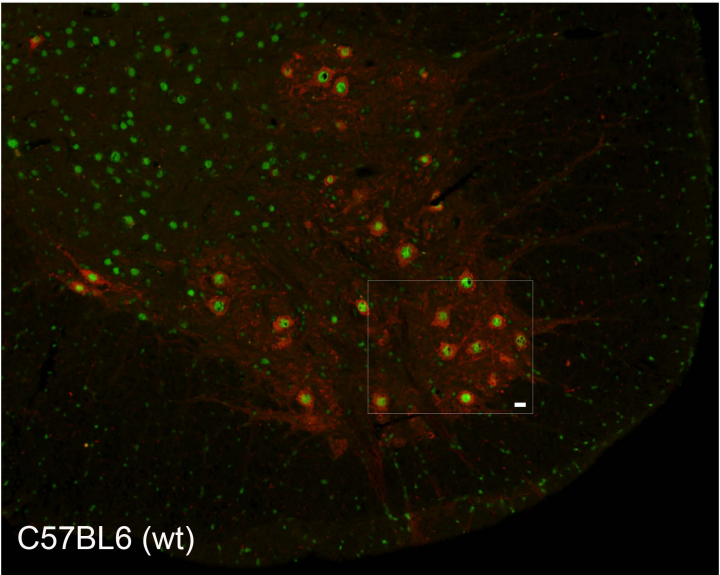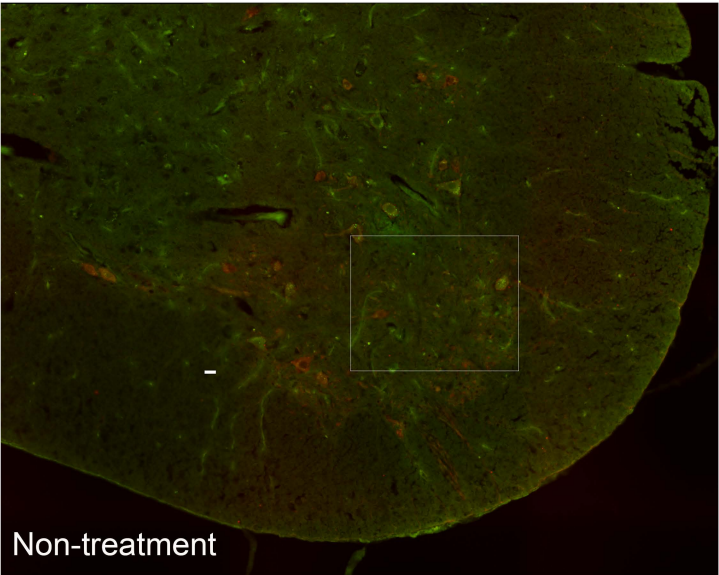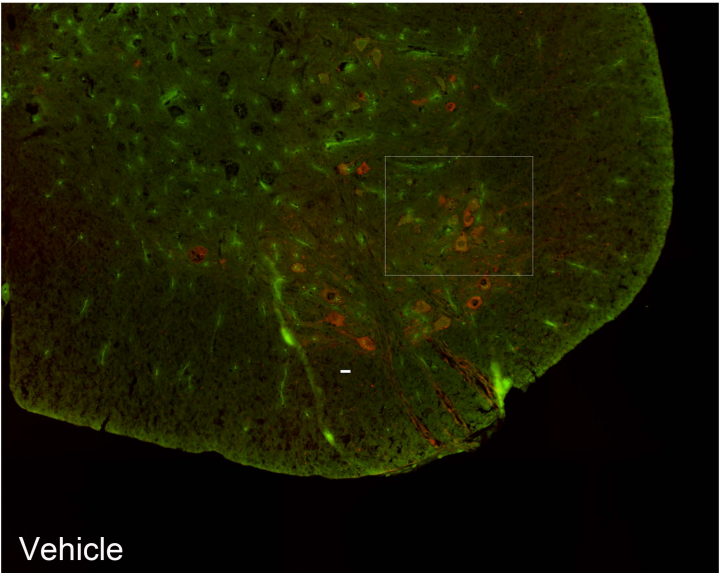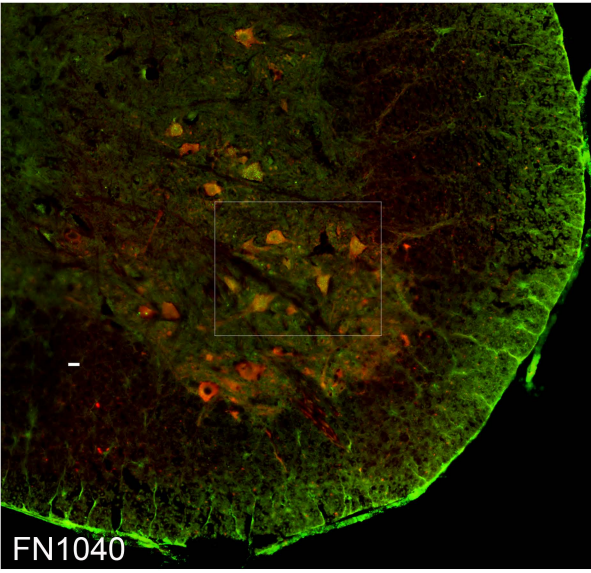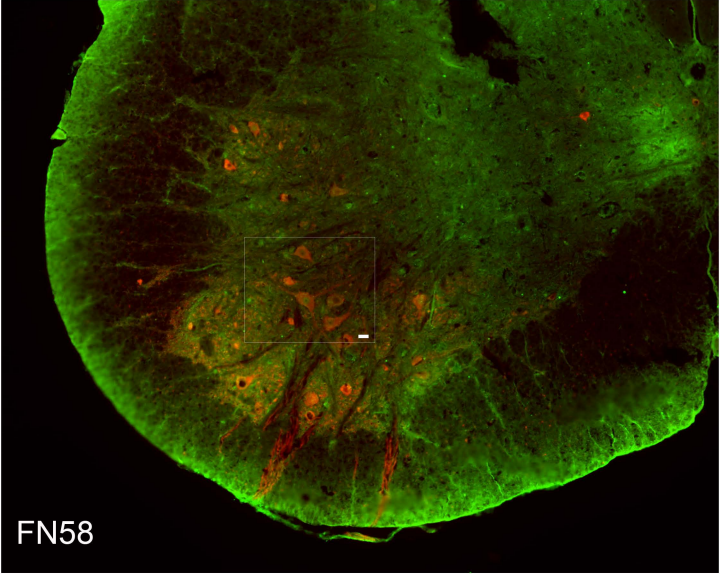

Supplement: Supplementary file 11 [file LSA-2021-01193_SdataF4.2.pdf]
